# Exome sequencing for disease gene discovery in Jeune’s Asphyxiating Thoracic Dystrophy

**DOI:** 10.1186/2046-2530-1-S1-P105

**Published:** 2012-11-16

**Authors:** P Taylor, S Wu, SF Nelson, DH Cohn, D Krakow

**Affiliations:** 1 Human Genetics, University of California Los Angeles, USA; 2 Orthopaedic Surgery, University of California Los Angeles, USA

## 

Asphyxiating Thoracic Dystrophy (ATD) is a clinically heterogeneous, autosomal recessive skeletal dysplasia characterized primarily by an abnormally narrow thorax with short ribs and short limbs. It has an estimated incidence of ~1 out of every 130,000 live births. Most patients die in early infancy or early childhood from respiratory insufficiency due to a small thorax. To date, four genes and one additional loci have been found to cause ATD in an autosomal recessive fashion. All four known genes are essential for normal primary cilia function. We employed the use of exome sequencing of seven unrelated patients affected with ATD to identify candidate mutations responsible for or contributing to the disorder. To maximize the success of gene discovery, we used Agilent’s SureSelect Exome Pull Down capturing 50 Mb of the protein-coding exome. Each sample library was sequenced using a single lane of Illumina HiSeq. We generated 252Gb of paired-end sequence data with a mean coverage of 115X per sample. The sequencing data was aligned using Novoalign and variants were called using the Genome Analysis Toolkit. Variants were filtered against dbSNP135, 69 complete Genomics samples, 95 NIEHS EGP exome samples, 5400 exomes from the NHLBI Exome Sequencing Project, and 40 in-house exome samples. Only mutations that were present in the above controls in the homozygous state were removed from the candidate list. After filtering, potential disease-causing mutations representing two known genes and four novel genes were found in all seven individuals.

